# The shrinking health advantage: unintentional injuries among children and youth from immigrant families

**DOI:** 10.1186/s12889-017-4612-1

**Published:** 2017-08-01

**Authors:** Natasha Ruth Saunders, Alison Macpherson, Jun Guan, Lisa Sheng, Astrid Guttmann

**Affiliations:** 1 The Hospital for Sick Children, 555 University Avenue, Toronto, Ontario M5G 1X8 Canada; 2 Department of Pediatrics, University of Toronto, Toronto, Canada; 3 Institute for Clinical Evaluative Sciences, Toronto, Canada; 4 Child Health Evaluative Sciences, SickKids Research Institute, Toronto, Canada; 5 York University, Toronto, Canada; 6 Dalla Lana School of Public Health, Institute of Health Policy, Management and Evaluation, The University of Toronto, Toronto, Canada

**Keywords:** Immigration, Pediatric, Migration

## Abstract

**Background:**

Immigrants typically arrive in good health. This health benefit can decline as immigrants adopt behaviours similar to native-born populations. Risk of injury is low in immigrants but it is not known whether this changes with increasing time since migration. We sought to examine the association between duration of residence in Canada and risk of unintentional injury.

**Methods:**

Population-based cross-sectional study of children and youth 0 to 24 years in Ontario, Canada (2011-2012), using linked health and administrative databases. The main exposure was duration of Canadian residence (recent: 0–5 years, intermediate: 6–10 years, long-term: >10 years). The main outcome measure was unintentional injuries. Cause-specific injury risk by duration of residence was also evaluated. Poisson regression models estimated rate ratios (RR) for injuries.

**Results:**

999951 immigrants were included with 24.2% recent and 26.4% intermediate immigrants. The annual crude injury rates per 100000 immigrants were 6831 emergency department visits, 151 hospitalizations, and 4 deaths. In adjusted models, recent immigrants had the lowest risk of injury and risk increased over time (RR 0.79; 95% CI 0.77, 0.81 recent immigrants, RR 0.90; 95% CI 0.88, 0.92 intermediate immigrants, versus long-term immigrants). Factors associated with injury included young age (0-4 years, RR 1.30; 95% CI 1.26, 1.34), male sex (RR 1.52; 95% CI 1.49, 1.55), and high income (RR 0.93; 95% CI 0.89, 0.96 quintile 1 versus 5). Longer duration of residence was associated with a higher risk of unintentional injuries for most causes except hot object/scald burns, machinery-related injuries, non-motor vehicle bicycle and pedestrian injuries. The risk of these latter injuries did not change significantly with increasing duration of residence in Canada. Risk of drowning was highest in recent immigrants.

**Conclusions:**

Risk of all-cause and most cause-specific unintentional injuries in immigrants rises with increasing time since migration. This indicates the need to develop strategies for maintaining the immigrant health advantage over time while balancing the desire to support integration, active living, and healthy child development.

**Electronic supplementary material:**

The online version of this article (doi:10.1186/s12889-017-4612-1) contains supplementary material, which is available to authorized users.

## Background

With the proliferation of global migration, increasing attention is being paid to health outcomes of immigrants in developed countries. Immigrants tend to arrive with better health than their native born counterparts. This healthy immigrant effect has been demonstrated across multiple health systems, in several countries, and for a number of conditions [1–7]. With increasing duration of residence in a host country, this health benefit can shrink as immigrants adopt behaviours and exposures more similar to the native population. This latter observation, demonstrated in adult populations, has been termed the “convergence hypothesis” [4, 8, 9].

The notion of the healthy immigrant effect has been less well studied in children and young adults but has been shown for immunizations [10], mental health [1, 11], and substance use [1]. The convergence hypothesis, however, has not been evaluated at a population-based level for pediatric health outcomes. Moreover, in adults, there is considerable variation in the effects of immigration on health and its subsequent convergence towards the native population depending on the health outcome and region of origin [8, 9].

Injury remains one of the leading causes of death, hospitalization, and emergency department (ED) visits for children and youth in North America and Europe [12, 13]. Most injuries are preventable, and injury reduction strategies, when targeted and implemented appropriately, are successful [14–16]. Immigrants have a lower risk of unintentional injury than Canadian born children [17–19]. Though there is some cause-specific variability, this has been demonstrated across all causes of injury [19]. The evidence for the extent to which the lower risk of injury persists with increasing duration of residence in a new country for children and young adults from immigrant families is not known. As immigrant populations acculturate, their risk-taking behaviours or exposures may change. Investigating patterns of injury across immigrant populations with increasing time since migration can help provide a more comprehensive picture of contributing factors to injury risk in this large and growing population. With better understanding of unintentional injury risk in immigrants, strategies can be developed for injury prevention, education, behaviour modification, and policy development so that immigrants can maintain their health advantage.

We sought to examine patterns of unintentional injury in children and youth from immigrant families in a large, diverse Canadian province with a single payer health care system where 20% of the population are immigrants [20]. Our specific objectives were to describe the epidemiology of unintentional injury-related emergency department visits, hospitalizations, and deaths overall and according to cause of injury in recent, intermediate-term, and long-term children and youth from immigrant families and by region of origin; and to test the association of unintentional injury and duration of residence and region of origin in children from immigrant families in Ontario, Canada.

## Methods

### Study Design

**Fig. 1:**
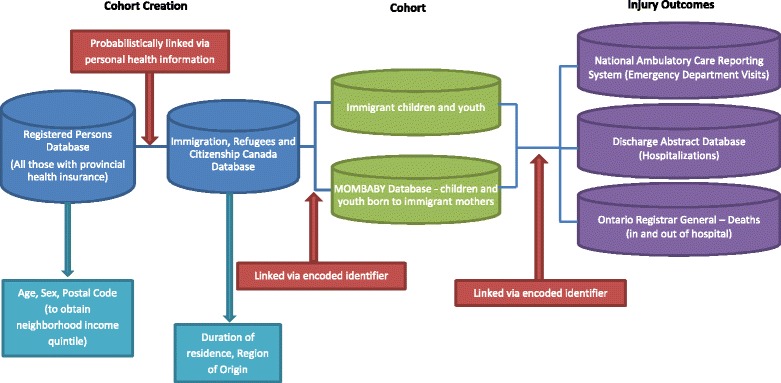
Data sources available and linked at the Institute for Clinical Evaluative Sciences

This population-based cross-sectional study was carried out at the Institute for Clinical Evaluative Sciences (ICES) using multiple, linked health and administrative databases (Fig. 1) through a research agreement with the Ontario Ministry of Health and Long-Term Care. Research ethics board approval was obtained from The Hospital for Sick Children and Sunnybrook Health Sciences Centre in Toronto, Ontario.

### Dataset Sources

The Ontario Health Insurance Plan (OHIP) is the single-payer, universal funding plan for medically necessary physician and hospital services in Ontario. Permanent residents admitted to Canada as immigrants or refugees are typically eligible for OHIP within 3 months of living in Ontario. OHIP eligible persons are entered into Ontario’s health care registry, the Registered Persons Database (RPDB), which contains socio-demographic information about residents including their age, sex, and postal code. Their OHIP number is encoded and linked to a number of other health administrative and demographic databases. The Canadian Institute for Health Information (CIHI) Discharge Abstract Database (DAD) and National Ambulatory Care Reporting System (NACRS) provide standard data collection and reporting tools to capture hospitalization and emergency department visit information, respectively, and include the main diagnosis. Linkage rates to the RPDB are greater than 97% [21]. These were used to obtain injury-related information where a hospital or ED visit occurred. The Ontario Registrar General – Death (Vital Stats) was used to identify in and out of hospital injury-related deaths in Ontario. Neighbourhood income quintile was obtained using Statistics Canada’s Postal Code Conversion File to link a patient’s postal code at the dissemination area level (400 to 700 persons) based on the 2006 Canadian Census [22].

The Permanent Resident Data System, a federal database maintained by Immigration, Refugees and Citizenship Canada (IRCC), holds socio-demographic and immigration information on all permanent residents landing in Ontario from January 1^st^, 1985 to the present. Permanent residents are immigrants who have been granted to the right to stay and work in Canada without limitations on their stay. It does not include data on temporary or undocumented immigrants. Information is collected from official immigration documents upon landing. The MOMBABY database is an ICES derived database that pairs all mothers with their newborns, delivered in hospital in Ontario through linkage of the CIHI-DAD inpatient admission records of delivering mothers and their newborns [21]. This was used to identify newborns born in Canada to immigrant mothers.

### Study Population

The study population included children and youth from birth to 24 years of age who were immigrants, or were born in Canada to immigrant mothers. To be included, individuals must have had an IRCC permanent resident record or be the child of a mother with an IRCC record and have a linked mother-baby dyad in the ICES MOMBABY database. Maternal immigrant status was included to reflect the influence of family immigrant status, rather than that of only the child. Parental health and risk-taking behaviours are particularly important for a child’s risk of injury (e.g. safe sleeping and cooking environments, car seat safety, etc.) Moreover, maternal immigrant status is associated with risk of injury in children [19, 23]. Individuals must have been residing in Ontario with a valid Ontario Health Insurance Plan (OHIP) number between January 1^st^ 2011 and December 31^st^, 2012.

### Outcome Measures

The main outcome measure was an unintentional injury-related visit to an ED or a hospitalization, or an unintentional injury related death (in and out of hospital) during the study window (2011 to 2012). The International Classification of Disease 10-CM External Cause of Injury Codes were used to identify and group injuries by cause [24]. Multiple events by the same patient were included although only one event per patient per day was included. Where individuals had an ED visit with a subsequent hospitalization, only the hospitalization was counted. Duplicate, overlapping, or transferred ED visits were not double counted so that only the first record for any given injury event was considered.

### Exposure Variables

The main exposure was duration of residence in Canada. Immigrants were grouped as recent (0 to 5 years in Canada), intermediate-term (6 to 10 years in Canada), and long-term (greater than 10 years in Canada). Duration of residence was determined from the difference of the number of years from the date of landing to December 31^st^, 2011 (study mid-point). If the patient was born in Canada to an immigrant mother, the duration of residence was calculated based on the maternal immigration information. The secondary exposure was region of origin, based on the country of birth, using modified IRCC region groupings [20].

### Covariates

A number of covariates that have been shown to be associated with risk of unintentional injury were considered. Older age (adolescence) is a strong predictor of injury risk, as is male sex [17, 25]. Both age and sex were therefore included as covariates. In a number of populations, low income has been associated with an increased risk of both unintentional and intentional injury [23, 26]. We therefore included postal code based neighbourhood income quintiles within dissemination areas (areas of 400 to 700 individuals) from health records as a covariate.

### Statistical Analysis

Descriptive statistics were performed for the main independent variables, outcome variables, and covariates. The crude injury rates were calculated, as were the total number of events. To compare outcomes of immigrants by duration of residence, multiple variable Poisson regression models adjusting for overdispersion were used to compute rate ratios with 95% confidence intervals. A sensitivity analysis excluding children and youth of immigrant mothers was also performed. For each model, variables were selected *a priori* and included in the regression analysis. The cause-specific rate ratios of injury by duration of residence was also calculated using multiple variable Poisson regression models adjusting for overdispersion. All statistical modelling was carried out using SAS Enterprise Guide, version 6.1 (SAS Institute Inc., Cary, NC).

## Results

There were 999 951 children and youth included in the study. Compared with long-term immigrants, recent immigrants had a greater proportion of young and lower neighbourhood income quintile individuals (Table 1). Most (49.5%) immigrants were from South and East Asia. The annual crude rates of unintentional injuries per 100 000 were 6830 for ED visits, 151 for hospitalizations, and 4 deaths.

**Table 1:** Children and youth from immigrant families in Ontario by duration of residence, 2011 to 2012

	Overall		Recent		Intermediate		Long-term	
	N	%	N	%	N	%	N	%
Overall	999951	100.0	242117	24.2	264453	26.4	493381	49.3
Age (years)								
0-4	227965	22.8	77930	32.2	72572	27.4	77463	15.7
5-9	197813	19.8	39394	16.3	64870	24.5	93549	19.0
10-14	199438	19.9	40005	16.5	42240	16.0	117193	23.8
15-19	198308	19.8	39465	16.3	43319	16.4	115524	23.4
20-24	176427	17.6	45323	18.7	41452	15.7	89652	18.2
Sex								
Female	486604	48.7	119032	49.2	127978	48.4	239594	48.6
Male	513347	51.3	123085	50.8	136475	51.6	253787	51.4
Income quintile								
Q1-lowest income	290151	29.0	90836	37.5	77208	29.2	122107	24.7
Q2	216221	21.6	52924	21.9	57026	21.6	106271	21.5
Q3	201353	20.1	42239	17.4	53734	20.3	105380	21.4
Q4	177852	17.8	34777	14.4	48029	18.2	95046	19.3
Q5-highest income	114374	11.4	21341	8.8	28456	10.8	64577	13.1
Source region								
E.Asia/Pacific	226268	22.6	56714	23.4	61623	23.3	107931	21.9
S.Asia	268939	26.9	68532	28.3	88598	33.5	111809	22.7
E.Europe/Central Asia	75865	7.6	14075	5.8	23425	8.9	38365	7.8
Africa	90135	9.0	23712	9.8	20981	7.9	45442	9.2
Middle East	95966	9.6	31668	13.1	26491	10.0	37807	7.7
S.America	48259	4.8	10555	4.4	11573	4.4	26131	5.3
Central America	84007	8.4	15796	6.5	12035	4.6	56176	11.4
US/UK/Western Europe	110178	11.0	21034	8.7	19693	7.4	69451	14.1
Missing	334	0.0	31	0.0	34	0.0	269	0.1

Table 2 shows the rates of unintentional-injury events by duration of residence and region of origin. Compared with recent immigrants, immigrants living in Canada for more than 10 years had higher rates of ED visits and hospitalizations for injuries across all age groups, income quintiles, and regions of origin with the exceptions of hospitalizations among 10- to 14-year-olds and Central Americans and emergency department visits among those from South America where rates were relatively unchanged. Immigrants from East and South Asia had the lowest rates of unintentional injury, and those from the United States/United Kingdom/Europe, Central Asia, and South America had the highest rates.

**Table 2:** Unintentional injury related emergency department (ED) visits, hospitalizations, and deaths by duration of residence, annualized 2011 to 2012

	ED visits			Hospitalizations			Deaths^a^		
	Recent	Intermediate	Long-term	Recent	Intermediate	Long-term	Recent	Intermediate	Long-term
Total events	12832	16610	35494	304	369	764	11	10	21
	Rate per 100 000 immigrants			Rate per 100 000 immigrants			Rate per 100 000 immigrants		
Overall Injury Rate	5866.2	6521.9	7437.0	138.8	144.9	160.1	4.8	3.9	4.3
Age (years)									
0-4	7133.4	7985.5	8824.0	164.9	163.4	214.7	-	-	-
5-9	5043.1	5886.6	6552.4	137.9	164.8	157.0	-	-	-
10-14	5579.1	6134.3	7523.5	134.0	96.2	131.3	-	-	-
15-19	5594.0	5856.1	7295.8	131.5	142.4	155.4	-	-	-
20-24	4922.2	6191.5	7311.2	104.1	134.9	163.9	-	-	-
Sex									
Female	4678.6	5187.1	5877.2	93.8	103.8	115.4	-	-	3.9
Male	7006.1	7774.1	8910.3	181.9	183.4	202.3	-	-	4.7
Income quintile									
Q1-lowest income	5802.5	6280.4	7315.0	153.9	152.5	184.1	-	-	5.1
Q2	5599.9	6126.3	7068.5	129.6	135.8	152.8	-	-	-
Q3	5591.8	6356.9	7328.0	132.7	147.9	149.7	-	-	-
Q4	6323.5	6951.5	7697.8	133.7	138.6	151.8	-	-	-
Q5-highest income	6554.3	7497.6	8053.0	118.6	147.6	156.4	0.0	-	-
Source region									
E.Asia/Pacific	4309.0	5289.1	5087.7	107.9	110.2	122.3	-	-	-
S.Asia	5245.8	5530.0	6544.3	152.3	154.1	164.2	-	-	-
E.Europe/Central Asia	7647.7	8423.2	9117.2	159.6	157.5	186.5	-	-	-
Africa	6307.7	7524.4	8156.2	140.9	148.8	169.5	-	-	-
Middle East	6316.2	7139.4	7708.5	146.0	154.1	131.1	-	-	-
S.America	8466.5	8423.1	8408.3	134.9	171.3	197.7	-	0.0	-
Central America	6912.7	8101.4	8404.1	189.1	174.5	171.5	-	-	-
US/UK/W. Europe	7513.8	8592.6	9852.4	115.4	146.2	184.6	-	-	-
Missing	10344.8	3225.8	9807.7	0.0	0.0	0.0	0.0	0.0	0.0

^a^Death rates for subgroups not reported due to small cell sizes. Institutional policy requires suppression of cell sizes <6 or where back-calculation could allow for identification of cell sizes < 6

In the multiple variable Poisson regression model (Table 3), after adjusting for age, sex, income quintile, and region of origin, recent immigrants had significantly lower rates of unintentional injury and this risk increased over time (rate ratio [RR] = 0.79, 95% confidence interval [CI] 0.77, 0.81 for recent immigrants, RR = 0.90; 95% CI 0.88, 0.92 for intermediate immigrants compared to long-term immigrants). Sensitivity analysis excluding children and youth born to immigrant mothers (Additional file 1: Tables S1 and S2), excluding infants less than one year (Additional file 2: Tables S3 and S4), and excluding those with missing region of origin (Additional file 3: Table S5) showed the direction of the effects of duration of residence did not change. Predictors of unintentional injury included young age (0 to 4 years, RR = 1.30; 95% CI 1.26, 1.34) and male sex (RR = 1.52; 95% CI 1.49, 1.55). Higher income quintile was associated with increased risk of unintentional injury. The risk of injury was lowest in immigrants from Asia (RR = 0.55; 95% CI 0.53, 0.57 for East Asia/Pacific, RR = 0.67; 95% CI 0.64, 0.69 for South Asia).

**Table 3:** Rate ratios of unintentional injuries in children and youth aged 0-24 years by duration of residence, 2011-2012

Duration of residence	Unadjusted rate ratios (95% CI)	Adjusted^a^ rate ratio (95% CI)
Recent	0.79 (0.75, 0.83)	0.79 (0.77, 0.81)
Intermediate	0.88 (0.84, 0.92)	0.90 (0.88, 0.92)
Longer-term (reference)	1	1
Age		
00-04	1.24 (1.16, 1.32)	1.30 (1.26, 1.34)
05-09	0.94 (0.88, 1.00)	0.94 (0.91, 0.98)
10-14	1.06 (0.99, 1.13)	1.05 (1.01, 1.08)
15-19	1.03 (0.96, 1.10)	1.01 (0.98, 1.05)
20-24 (reference)	1	1
Sex		
Male	1.51 (1.46, 1.57)	1.52 (1.49, 1.55)
Female (reference)	1	1
Income		
Q1	0.87 (0.81, 0.93)	0.93 (0.89, 0.96)
Q2	0.85 (0.79, 0.91)	0.91 (0.88, 0.94)
Q3	0.88 (0.82, 0.95)	0.92 (0.89, 0.96)
Q4	0.95 (0.88, 1.02)	0.97 (0.94, 1.01)
Q5-highest income (reference)	1	1
Source Regions		
East Asia and Pacific	0.54 (0.51, 0.58)	0.55 (0.53, 0.57)
South Asia	0.65 (0.61, 0.68)	0.67 (0.64, 0.69)
Eastern Europe/Central Asia	0.94 (0.88, 1.01)	0.95 (0.91, 0.99)
Africa	0.82 (0.77, 0.88)	0.85 (0.81, 0.88)
Middle East	0.77 (0.72, 0.83)	0.81 (0.78, 0.84)
South America	0.92 (0.85, 1.00)	0.94 (0.89, 0.98)
Central America	0.88 (0.83, 0.95)	0.89 (0.85, 0.93)
Missing	0.98 (0.44, 2.20)	0.97 (0.60, 1.56)
US/UK/Western Europe (reference)	1	1

^a^Adjusted for age, sex, neighbourhood income quintile, and source region

**Fig. 2:**
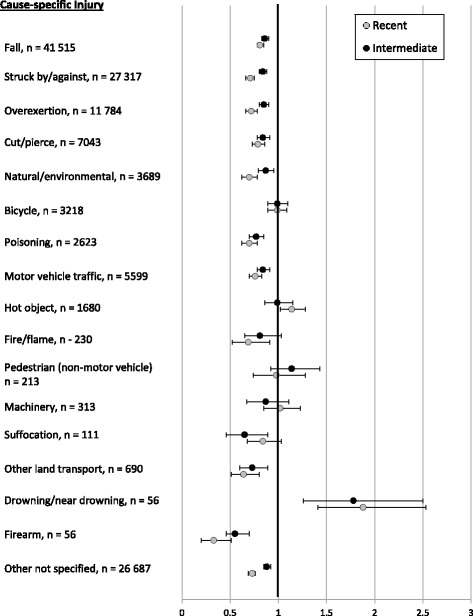
Adjusted rate ratios for cause-specific injuries by duration of residence. (Reference: Long-term immigrant)

Figure 2 shows the cause-specific risk of unintentional injury by duration of residence in Canada. Longer duration of residence was associated with a higher risk of unintentional injuries for most causes except hot object/scald burns, machinery-related injuries, non-motor vehicle bicycle and pedestrian injuries. The risk of these latter injuries did not change significantly with increasing duration of residence in Canada. Moreover, risk of injury was highest in recent immigrants for drowning. In evaluating cause-specific injury by severity (i.e. those requiring hospitalization or causing death versus ED visit), similar patterns by duration of residence were observed in cause-specific rates of injury (Table 4).

**Table 4:** Unintentional injury related emergency department (ED) visits, hospitalizations, and deaths by duration of residence and mechanism of injury, 2011 to 2012^a^

	ED visits, per 100 000 immigrants			Hospitalizations, per 100 000 immigrants			Deaths, per 100 000 immigrants		
	Recent	Intermediate	Long-term	Recent	Intermediate	Long-term	Recent	Intermediate	Long-term
Falls	1988.9	2105.8	2195.3	56.9	62.6	59.2	0.0	0.0	-
Struck by/against	1081.9	1300.5	1641.8	11.2	11.8	18.4	-	-	-
Overexertion	467.9	538.3	725.3	-	3.7	4.6	0.0	0.0	0.0
Cut/pierce	314.5	323.4	416.1	-	-	3.7	0.0	0.0	0.0
Natural/environmental	151.8	193.8	208.4	-	2.9	2.7	0.0	0.0	0.0
Bicycle, no-motor vehicle	144.5	157.3	174.2	4.6	7.1	6.9	0.0	0.0	0.0
Poisoning	118.4	121.9	132.6	10.3	7.3	12.8	-	0.0	-
Motor vehicle traffic	224.9	237.4	328.4	12.3	11.8	13.6	-	-	1.5
Hot object/scald	109.0	85.6	71.0	5.7	5.3	3.9	0.0	0.0	0.0
Fire/flame	9.1	10.6	13.1	-	-	-	0.0	0.0	0.0
Pedestrian (non-motor vehicle)	9.4	11.2	9.6	-	-	1.2	-	0.0	-
Machinery	16.5	13.0	17.1	-	-	-	0.0	0.0	0.0
Suffocation	4.3	4.7	3.7	2.7	0.0	1.6	-	-	-
Other land transport	22.4	25.5	43.9	-	-	2.5	0.0	-	-
Drowning	-	-	1.2	-	-	-	-	-	-
Firearm	-	-	2.9	0.0	-	1.3	0.0	0.0	0.0
Other or not specified	1200.3	1389.6	1452.4	24.5	24.9	26.4	-	-	-

^a^Rates for some subgroups not reported due to small cell sizes. Institutional policy requires suppression of cell sizes <6 or where back-calculation could allow for identification of cell sizes < 6

## Discussion

In this large population-based cross-sectional study, we report longer duration of residence in Canada is associated with an increased risk of unintentional injuries in children and young adults from immigrant families. This was observed across most causes of injury, except for drowning and scald burns where the highest rates of injury were among newcomers (≤ 5 years in Canada). Risk of unintentional injury was lowest in immigrants from South and East Asia and highest in those from Europe and the Americas.

The rates of unintentional injury in our cohort of immigrants are much lower than published rates for pediatric injuries in the general child and youth population of Ontario [17], supporting a healthy immigrant effect. Our study is the first to report a loss of this health advantage over time, with immigrant injury risk converging towards levels of the general Canadian population. This is particularly important given the amenability of injury risk to appropriately targeted safety interventions [13].

The shrinking health benefit of immigrants over time may be explained by a number of factors. As immigrants acculturate, they may introduce changes to their parenting style, including direct supervision of children. With settlement and integration, children and youth may have increased exposures such as more time spent in motor vehicles with less time spent exploring urban areas as pedestrians. The worsening injury rates in immigrants may reflect adoption of unhealthy, risk taking behaviours known to contribute to injury, such as drug and alcohol use [13] or involvement with gang-related firearm violence [27, 28]. There may also be changes in access to primary care, which has been shown to be worse over time from migration [29]. This may reduce opportunities for preventative counselling on injuries and safe play over time in Canada. Similarly, barriers to care-seeking including initial distrust in or use of the health care system may preclude recent immigrants from visiting a hospital for injuries. Conversely, our findings of increasing falls, cuts, and overexertion may indicate improved access to play spaces with increased physical activity or participation in sport by immigrants as they integrate into life in Canada.

The observed rates of cause-specific injuries by duration of residence are not unexpected. Research from Denmark has shown that while pediatric immigrants have lower risk of injury, a higher proportion scald and hot oil burns occurred in immigrants compared with native Danes [23]. More time spent at home cooking and preparing foods by immigrants, especially within newcomers, may explain this finding. Drowning rates among newcomers may be related to lack of knowledge about safe swim environments and highlights an area in need of intervention. Relatively high rates of suffocation were observed in recent immigrants. Recent immigrants may have inferior understanding of North American or European product safety guidelines such as crib safety (placing infant supine, avoiding drop-side rails, and not using loose bedding in cribs or blind cord safety), all of which can lead to entrapment or strangulation and subsequent suffocation [13]. Understanding the variability in the influence of duration of residence over time on cause-specific injuries will be important to study as injury prevention strategies are developed. For example, the unchanged rates of bicycle, pedestrian, and machinery injuries may reflect the countering protective effects of being a recent immigrant and changes in exposures over time in Canada. This remains to be studied.

We observed significant differences in risk of injury by region of origin. In particular, South and East Asian immigrant populations had very low rates of injury. Others have described significant variability in rates of injury mortality by region of origin [5] though comparisons to Asian immigrant populations have not previously been reported. Interestingly, South and East Asian immigrants have among the highest in-country unintentional injury rates across the globe (49.0 injuries per 100 000 population), with rates second only to African countries [13]. Clearly, this risk is not brought with them through immigration. Our observations may be related to selection factors in those who immigrate to Canada as well as cultural differences in risk taking behaviours and exposures that reduce overall risk of unintentional injury upon arrival in Canada.

### Strengths and Limitations

This is the first study to report injury risk by duration of residence in a host country. The large sample size, which included the vast majority of immigrants to Ontario, and the use of health services data to ascertain an injury event sets this study apart from other injury studies which have much smaller sample sizes [30–32] or rely on self-report which can bias results, especially for injury reporting [33]. The findings may be generalizable to many developed regions including the United States, Australia, and the European Union where large proportions of the population are foreign born, from a wide variety of countries, with similar immigration policies and comparable safety standards. However, there are a number of important limitations to our study. As it is based on registry data, this precludes our understanding of circumstances that may have contributed to injury events (i.e. supervision, intoxication, behavioural co-morbidities, etc.). Other demographic information such as family education level or paternal immigrant status may have also helped to better understand injury risk over time. Our database does not include temporary or undocumented immigrants and may not be generalizable to these groups. Immigrants who migrate to Ontario after landing in another province (approximately 10%) are misclassified as non-immigrants and are not included in our study. Finally, linkage of the IRCC database to RPDB is not perfect, with a higher proportion of non-linked immigrant populations from Asia, where common names make accurate linkage more challenging [27].

## Conclusions

The risk of all-cause and most cause-specific unintentional injuries in immigrant children and youth rises with increasing duration of residence in Canada. This important finding indicates there is a need to understand why this is occurring and to develop strategies, which may include providing education about water and cooking safety for recent immigrants, to maintain the immigrant health advantage over time after migration. This must be done while balancing the desire to support active living and healthy child development.

## Additional files

Additional file 1: Table S1. Immigrant children and youth in Ontario by duration of residence, 2011 to 2012. **Table S2.** Rate ratios of unintentional injuries for immigrant children and youth aged 0-24 years by duration of residence, 2011-2012. Descriptive table of cohort of immigrants excluding children and youth born to immigrant mothers and table of adjusted rate ratios testing the association of duration of residency in Canada and risk of unintentional injury, excluding children and youth born to immigrant mothers. (DOCX 19 kb)

Additional file 2: Table S3. Children and youth from immigrant families in Ontario by duration of residence, 2011 to 2012. **Table S4.** Adjusted rate ratios of unintentional injuries in children aged 1-24 years by duration of residence, 2011-2012. Descriptive table of cohort of immigrants excluding children less than one year of age and table of adjusted rate ratios testing the association of duration of residency in Canada and risk of unintentional injury, excluding children less than one year of age. (DOCX 13 kb)

Additional file 3: Table S5. Adjusted rate ratios of unintentional injuries in children and youth aged 0-24 years by duration of residence, excluding those where region of origin is missing, 2011-2012. Sensitivity analysis testing the association of unintentional injuries in children and youth by duration of residence in Canada where those whose region of origin was missing was excluded from the analysis. (DOCX 22 kb)

## Abbreviations

CI: Confidence interval
CIHI: Canadian Institute for Health Information
DAD: Discharge Abstract Database
ED: Emergency department
ICES: Institute for Clinical Evaluative Sciences
IRCC: Immigration, Refugees and Citizenship Canada
MOHLTC: Ministry of Health and Long-Term Care
NACRS: National Ambulatory Care Reporting System
OHIP: Ontario Health Insurance Plan
RR: RATE RATIO
