# Supplementary material for: The shrinking health advantage: unintentional injuries among children and youth from immigrant families
Source: BMC Public Health. 2017 Aug 1;18:73. doi: 10.1186/s12889-017-4612-1 (PMC5540344; doi:10.1186/s12889-017-4612-1)
Supplement: Supplementary file 1 — Immigrant children and youth in Ontario by duration of residence, 2011 to 2012. Table S2. Rate ratios of unintentional injuries for immigrant children and youth aged 0-24 years by duration of residence, 2011-2012. Descriptive table of cohort of immigrants excluding children and youth born to immigrant mothers and table of adjusted rate ratios testing the association of duration of residency in Canada and risk of unintentional injury, excluding children and youth born to immigrant mothers. (DOCX 19 kb) [file 12889_2017_4612_MOESM1_ESM.docx]

| **Table S1.** Immigrant children and youth in Ontario by duration of residence, 2011 to 2012. | | | | | | | | |
| --- | --- | --- | --- | --- | --- | --- | --- | --- |
|  | **Overall** | | **Recent** | | **Intermediate** | | **Long-tem** | |
|  | **N** | **%** | **N** | **%** | **N** | **%** | **N** | **%** |
| **Overall** | 436676 | 100.0 | 178909 | 41.0 | 136675 | 31.3 | 121092 | 27.7 |
| **Age (years)** |  |  |  |  |  |  |  |  |
| 0-4 | 15590* | 3.6 | 15590* | 32.2 | <=5 | 0.0 | 0 | 0.0 |
| 5-9 | 49160* | 11.3 | 38530* | 16.3 | 10630* | 7.8 | <=5 | 0.0 |
| 10-14 | 90511 | 20.7 | 40005 | 16.5 | 41280* | 30.2 | 9210* | 7.6 |
| 15-19 | 122083 | 28.0 | 39464 | 16.3 | 43315 | 31.7 | 39304 | 32.5 |
| 20-24 | 159336 | 36.5 | 45322 | 18.7 | 41448 | 30.2 | 72566 | 59.9 |
| **Sex** |  |  |  |  |  |  |  |  |
| Female | 212998 | 48.8 | 88305 | 49.2 | 65707 | 48.1 | 58986 | 48.7 |
| Male | 223678 | 51.2 | 90604 | 50.8 | 70968 | 51.9 | 62106 | 51.3 |
| **Income quintile** |  |  |  |  |  |  |  |  |
| Q1-lowest income | 138246 | 31.7 | 68272 | 37.5 | 40249 | 29.4 | 29725 | 24.75 |
| Q2 | 92634 | 21.2 | 38469 | 21.9 | 28928 | 21.2 | 25237 | 20.8 |
| Q3 | 83164 | 19.0 | 30800 | 17.4 | 27440 | 20.1 | 24924 | 20.6 |
| Q4 | 74444 | 17.0 | 25304 | 14.4 | 24873 | 18.2 | 24267 | 20.0 |
| Q5-highest income | 48188 | 11.0 | 16064 | 8.8 | 15185 | 11.1 | 16939 | 14.0 |
| **Source region** |  |  |  |  |  |  |  |  |
| E.Asia/Pacific | 97175 | 22.3 | 42277 | 23.6 | 29936 | 21.9 | 24962 | 20.6 |
| S.Asia | 112546 | 25.8 | 45112 | 25.2 | 41089 | 30.1 | 26345 | 21.8 |
| E.Eur./Cent. Asia | 37982 | 8.7 | 9797 | 5.5 | 12951 | 9.5 | 15234 | 12.6 |
| Africa | 36942 | 8.5 | 17685 | 9.9 | 10248 | 7.5 | 9009 | 7.4 |
| Middle East | 58412 | 13.4 | 26229 | 14.7 | 17498 | 12.8 | 14685 | 12.1 |
| S.America | 19071 | 4.4 | 8041 | 4.5 | 6274 | 4.6 | 4756 | 3.9 |
| Central America | 27347 | 6.3 | 12437 | 7.0 | 6280 | 4.6 | 8630 | 7.1 |
| US/UK/West.Eur. | 47082 | 10.8 | 17302 | 9.7 | 12377 | 9.1 | 17403 | 14.4 |
| Missing | 119 | 0.0 | 29 | 0.0 | 22 | 0.0 | 68 | 0.1 |

*= Numbers rounded to prevent re-identification of small cell sizes as per institutional policy.

|  |  | |  | |
| --- | --- | --- | --- | --- |
| **Table S2.** Rate ratios of unintentional injuries for immigrant children and youth aged 0-24 years by duration of residence, 2011-2012. | | | | |
| **Duration of Residence** | | **Unadjusted Rate Ratio**  **(95% CI)** | | **Adjusted* Rate Ratio**  **(95% CI)** |
| Recent | | 0.81 (0.76, 0.87) | | 0.87 (0.84, 0.90) |
| Intermediate | | 0.91 (0.85, 0.97) | | 0.96 (0.92, 0.99) |
| Longer-term (reference) | | 1 | | 1 |
| **Age** | |  | |  |
| 00-04 | | 1.00 (0.86, 1.17) | | 1.09 (1.00, 1.18) |
| 05-09 | | 0.83 (0.75, 0.91) | | 0.87 (0.82, 0.91) |
| 10-14 | | 0.96 (0.89, 1.04) | | 1.00 (0.96, 1.04) |
| 15-19 | | 0.95 (0.89, 1.02) | | 0.97 (0.93, 1.00) |
| 20-24 (reference) | | 1 | | 1 |
| **Sex** | |  | |  |
| Male | | 1.71 (1.64, 1.80) | | 1.72 (1.67, 1.77) |
| Female (reference) | | 1 | | 1 |
| **Income** | |  | |  |
| Q1 | | 0.89 (0.81, 0.97) | | 0.95 (0.90, 0.99) |
| Q2 | | 0.86 (0.78, 0.94) | | 0.92 (0.88, 0.97) |
| Q3 | | 0.88 (0.80, 0.97) | | 0.92 (0.87, 0.97) |
| Q4 | | 0.96 (0.87, 1.06) | | 0.98 (0.93, 1.03) |
| Q5-highest income (reference) | | 1 | | 1 |
| **Source Regions** | |  | |  |
| East Asia and Pacific | | 0.49 (0.45, 0.53) | | 0.50 (0.47, 0.52) |
| South Asia | | 0.65 (0.60, 0.70) | | 0.65 (0.62, 0.69) |
| Eastern Europe/Central Asia | | 1.09 (1.01, 1.19) | | 1.07 (1.02, 1.13) |
| Africa | | 0.90 (0.82, 0.98) | | 0.92 (0.87, 0.97) |
| Middle East | | 0.85 (0.79, 0.93) | | 0.86 (0.82, 0.91) |
| South America | | 1.06 (0.96, 1.18) | | 1.08 (1.01, 1.15) |
| Central America | | 0.97 (0.88, 1.07) | | 0.99 (0.93, 1.05) |
| Missing | | 1.17 (0.40, 3.46) | | 1.15 (0.59, 2.24) |
| US/UK/Western Europe (reference) | | 1 | | 1 |

*= Adjusted for age, sex, neighbourhood income quintile, and source region.
