# Supplementary material for: The shrinking health advantage: unintentional injuries among children and youth from immigrant families
Source: BMC Public Health. 2017 Aug 1;18:73. doi: 10.1186/s12889-017-4612-1 (PMC5540344; doi:10.1186/s12889-017-4612-1)
Supplement: Supplementary file 2 — Children and youth from immigrant families in Ontario by duration of residence, 2011 to 2012. Table S4. Adjusted rate ratios of unintentional injuries in children aged 1-24 years by duration of residence, 2011-2012. Descriptive table of cohort of immigrants excluding children less than one year of age and table of adjusted rate ratios testing the association of duration of residency in Canada and risk of unintentional injury, excluding children less than one year of age. (DOCX 13 kb) [file 12889_2017_4612_MOESM3_ESM.docx]

|  |  | |
| --- | --- | --- |
| **Table S5.** Adjusted* rate ratios of unintentional injuries in children and youth aged 0-24 years by duration of residence, excluding those where region of origin is missing, 2011-2012. | | |
| **Duration of Residence** | | **Rate Ratio (95% CI)** |
| Recent | | 0.79 (0.77, 0.82) |
| Intermediate | | 0.90 (0.88, 0.92) |
| Longer-term (reference) | | 1 |
| **Age** | |  |
| 00-04 | | 1.30 (1.26, 1.34) |
| 05-09 | | 0.94 (0.91, 0.98) |
| 10-14 | | 1.05 (1.01, 1.08) |
| 15-19 | | 1.01 (0.98, 1.05) |
| 20-24 (reference) | | 1 |
| **Sex** | |  |
| Male | | 1.52 (1.49, 1.55) |
| Female (reference) | | 1 |
| **Income** | |  |
| Q1 | | 0.93 (0.89, 0.96) |
| Q2 | | 0.91 (0.88, 0.94) |
| Q3 | | 0.92 (0.89, 0.96) |
| Q4 | | 0.97 (0.94, 1.01) |
| Q5-highest income (reference) | | 1 |
| **Source Regions** | |  |
| East Asia and Pacific | | 0.55 (0.53, 0.57) |
| South Asia | | 0.67 (0.64, 0.69) |
| Eastern Europe/Central Asia | | 0.95 (0.91, 0.99) |
| Africa | | 0.85 (0.81, 0.88) |
| Middle East | | 0.81 (0.78, 0.85) |
| South America | | 0.94 (0.89, 0.98) |
| Central America | | 0.89 (0.85, 0.93) |
| US/UK/Western Europe (reference) | | 1 |

*= Adjusted for age, sex, neighbourhood income quintile, and source region.
