# Supplementary material for: The shrinking health advantage: unintentional injuries among children and youth from immigrant families
Source: BMC Public Health. 2017 Aug 1;18:73. doi: 10.1186/s12889-017-4612-1 (PMC5540344; doi:10.1186/s12889-017-4612-1)
Supplement: Supplementary file 3 — Adjusted rate ratios of unintentional injuries in children and youth aged 0-24 years by duration of residence, excluding those where region of origin is missing, 2011-2012. Sensitivity analysis testing the association of unintentional injuries in children and youth by duration of residence in Canada where those whose region of origin was missing was excluded from the analysis. (DOCX 22 kb) [file 12889_2017_4612_MOESM2_ESM.docx]

| **Table S3.** Children and youth from immigrant families in Ontario by duration of residence, 2011 to 2012. | | | | | | | | | | | | |
| --- | --- | --- | --- | --- | --- | --- | --- | --- | --- | --- | --- | --- |
|  | **Overall** | | | | **Recent** | | **Intermediate** | | | **Long-term** | | |
|  | **N** | **%** |  | **N** | **%** |  | **N** |  | **%** | | **N** | **%** |
| **Overall** | 963646 | 100.0 |  | 225384 | 23.4 |  | 255542 |  | 26.5 | | 482720 | 50.0 |
| **Age (years)** |  |  |  |  |  |  |  |  |  | |  |  |
| 1-4 | 191660 | 19.9 |  | 61197 | 27.2 |  | 63661 |  | 24.9 | | 66802 | 13.8 |
| 5-9 | 197813 | 20.5 |  | 39394 | 17.5 |  | 64870 |  | 25.4 | | 93549 | 19.4 |
| 10-14 | 199438 | 20.7 |  | 40005 | 16.7 |  | 42240 |  | 16.5 | | 117193 | 24.3 |
| 15-19 | 198308 | 20.6 |  | 39465 | 17.5 |  | 43319 |  | 17.0 | | 115524 | 23.9 |
| 20-24 | 176427 | 18.3 |  | 45323 | 20.1 |  | 41452 |  | 16.2 | | 89652 | 18.6 |
| **Sex** |  |  |  |  |  |  |  |  |  | |  |  |
| Female | 469113 | 48.7 |  | 110911 | 49.2 |  | 123669 |  | 48.4 | | 234533 | 48.6 |
| Male | 494533 | 51.3 |  | 114473 | 50.8 |  | 131873 |  | 51.6 | | 248187 | 51.4 |
| **Income quintile** |  |  |  |  |  |  |  |  |  | |  |  |
| Q1-lowest income | 278667 | 28.9 |  | 84733 | 37.6 |  | 74572 |  | 29.2 | | 119362 | 24.7 |
| Q2 | 207937 | 21.6 |  | 49013 | 21.7 |  | 54960 |  | 21.5 | | 103964 | 21.5 |
| Q3 | 194210 | 20.2 |  | 39269 | 17.4 |  | 51799 |  | 20.3 | | 103142 | 21.4 |
| Q4 | 171850 | 17.8 |  | 32372 | 14.4 |  | 46585 |  | 18.2 | | 92893 | 19.2 |
| Q5-highest income | 110982 | 11.5 |  | 19997 | 8.9 |  | 27626 |  | 10.8 | | 63359 | 13.1 |
| **Source region** |  |  |  |  |  |  |  |  |  | |  |  |
| E.Asia/Pacific | 217159 | 22.5 |  | 52427 | 23.3 |  | 58838 |  | 23.0 | | 105894 | 21.9 |
| S.Asia | 258428 | 26.8 |  | 62659 | 27.8 |  | 85920 |  | 33.6 | | 109849 | 22.8 |
| E.Europe/Central Asia | 73176 | 7.6 |  | 13000 | 5.8 |  | 22693 |  | 8.9 | | 37483 | 7.8 |
| Africa | 86727 | 9.0 |  | 22120 | 9.8 |  | 20216 |  | 7.9 | | 44391 | 9.2 |
| Middle East | 93204 | 9.7 |  | 30236 | 13.4 |  | 25958 |  | 10.2 | | 37010 | 7.7 |
| S.America | 46556 | 4.8 |  | 9908 | 4.4 |  | 11170 |  | 4.4 | | 25478 | 5.3 |
| Central America | 81005 | 8.4 |  | 14928 | 6.6 |  | 11539 |  | 4.5 | | 54538 | 11.3 |
| US/UK/Western Europe | 10706 | 11.1 |  | 20075 | 8.9 |  | 19176 |  | 7.5 | | 67815 | 14.0 |
| Missing | 325 | 0.0 |  | 31 | 0.0 |  | 32 |  | 0.0 | | 262 | 0.1 |

|  |  | |
| --- | --- | --- |
| **Table S4.** Adjusted* rate ratios of unintentional injuries in children aged 1-24 years by duration of residence, 2011-2012. | | |
| **Duration of Residence** | | **Rate Ratio (95% CI)** |
| Recent | | 0.80 (0.78, 0.83) |
| Intermediate | | 0.90 (0.88, 0.92) |
| Longer-term (reference) | | 1 |
| **Age** | |  |
| 00-04 | | 1.50 (1.45, 1.55) |
| 05-09 | | 0.95 (0.91, 0.98) |
| 10-14 | | 1.05 (1.01, 1.08) |
| 15-19 | | 1.01 (0.98, 1.05) |
| 20-24 (reference) | | 1 |
| **Sex** | |  |
| Male | | 1.52 (1.49, 1.55) |
| Female (reference) | | 1 |
| **Income** | |  |
| Q1 | | 0.93 (0.90, 0.96) |
| Q2 | | 0.91 (0.88, 0.94) |
| Q3 | | 0.92 (0.89, 0.96) |
| Q4 | | 0.97 (0.94, 1.01) |
| Q5-highest income (reference) | | 1 |
| **Source Regions** | |  |
| East Asia and Pacific | | 0.55 (0.53, 0.57) |
| South Asia | | 0.67 (0.64, 0.69) |
| Eastern Europe/Central Asia | | 0.95 (0.91, 0.99) |
| Africa | | 0.85 (0.81, 0.88) |
| Middle East | | 0.81 (0.78, 0.84) |
| South America | | 0.94 (0.89, 0.98) |
| Central America | | 0.89 (0.86, 0.93) |
| Missing | | 0.98 (0.61, 1.59) |
| US/UK/Western Europe (reference) | | 1 |

*= Adjusted for age, sex, neighbourhood income quintile, and source region.
